# Socioeconomic Inequality and Its Determinants Regarding Infant Mortality in Iran

**DOI:** 10.5812/ircmj.17602

**Published:** 2014-06-05

**Authors:** Maryam Damghanian, Mohammad Shariati, Khadigeh Mirzaiinajmabadi, Masud Yunesian, Mohammad Hassan Emamian

**Affiliations:** 1 Department of Reproductive Health, Shahroud University of Medical Sciences, Shahroud, IR Iran; 2 Department of Community Medicine, School of Medicine, Tehran University of Medical Sciences, Tehran, IR Iran; 3 Department of Midwifery, School of Nursing and Midwifery, Mashhad University of Medical Sciences, Mashhad, IR Iran; 4 Department of Environmental Health Engineering, School of Public Health, Tehran University of Medical Sciences, Tehran, IR Iran; 5 Center for Health Related Social and Behavioral Sciences Research, Shahroud University of Medical Sciences, Shahroud, IR Iran

**Keywords:** Infant Mortality, Socioeconomic Factors, Iran, Inequality

## Abstract

**Background:**

Infant mortality rate is a useful indicator of health conditions in the society, the racial and socioeconomic inequality of which is from the most important measures of social inequality.

**Objectives:**

The aim of this study was to determine the socioeconomic inequality and its determinants regarding infant mortality in an Iranian population.

**Patients and Methods:**

This cross-sectional study was performed on 3794 children born during 2010-2011 in Shahroud, Iran. Based on children’s addresses and phone numbers, 3412 were available and finally 3297 participated in the study. A data collection form was filled out through interviewing the mothers as well as using health records. Using principal component analysis, the study population was divided to high and low socioeconomic groups based on the case’s home asset, education and job of the household’s head, marital status, and composition of the household members. Inequality between the groups with regard to infant mortality was investigated by Blinder-Oaxaca decomposition method.

**Results:**

The mortality rate was 15.1 per 1000 live births in the high socioeconomic group and 42.3 per 1000 in the low socioeconomic group. Mother's education, consanguinity of parents, and infant's nutrition type and birth weight constituted 44% of the gap contributing factors. Child's gender, high-risk pregnancy, and living area had no impact on the gap.

**Conclusions:**

There was considerable socioeconomic inequality regarding infant mortality in Shahroud. Mother's education was the most contributing factor in this inequality.

## 1. Background

Children are a vulnerable group in any society (1). Infant mortality rate is a useful indicator for the society health condition (2). One of the main millennium development goals is reduction of the under-five mortality rate by two thirds between 1990 and 2015 (3). Infant death constitutes a major percentage of the under-five mortality rate in the world; for instance, in 2010 it accounted for 70% of the under-five mortality, in a way that out of estimated 7.7 million under-five deaths, nearly 5.4 million were infants (4). Health is a basic human right (5). Health equality is a recognized right and is in line with ethical rules. Hence, all people have the right to access the highest levels of healthcare (6). Although it has been a long time since equality was set as a main objective of the healthcare sector, inequality still exists between the poor and the rich, such that the poor has a higher mortality rate. The poor uses fewer healthcare services while they need more (7). Studies have demonstrated that child mortality rate is considerably higher among low socioeconomic groups (1, 8). While mortality rate has noticeably decreased in many countries, the health inequality has further expanded among nations, regions and social classes over the past two decades (9). Racial and socioeconomic disparities leading to infant mortality are from the most important measures of social inequality (10). Reducing the health gap is a strategic and valuable objective of governments. Enhancing the health standards among low socioeconomic groups of people and eliminating health inequality between high and low socioeconomic classes have been the key objectives of certain international organizations like the World Bank and World Health Organization (WHO) (11). Acquiring reliable information about causes of death is a key step for health planning and setting budget priorities in health programs (12). Conducting studies on inequality-resulted health outcomes between different socioeconomic groups is gaining popularity among health researchers and policymakers (13). A range of measures are used for identifying inequality. Numerous researches have been conducted worldwide on inequality in different fields of health using these measures (14, 15) and some of them have studied inequality in child mortality (16). The measures only show the level of inequality; while, the important point after identifying inequality is to determine the factors that have contributed to this matter. For this purpose, decomposition methods are used (17).

## 2. Objectives

In the present study, we used the Blinder-Oaxaca method, which has been used less frequently in health studies, to describe determinants of infant mortality in Iran and investigate the socioeconomic inequality in this important health indicator.

## 3. Patients and Methods

In this cross sectional study, among 3794 children born during 2010-2011 in Shahroud, according to the existing addresses and phone numbers, 3412 children were available (89.9% access rate) and ultimately 3297 consented to enter the study (96.6% response rate). The sampling method was census. Shahroud is located in north-eastern part of Iran; it has an average socioeconomic status and similar infant mortality rate (21.8 per 1000 live births) to the national average (22 per 1000 live births). A data collection form was used for collecting the data. The form contained information about parents, home assets, prenatal care and labor, and infant's nutrition, vaccination and growth. Interviewers were trained in the Department of Health in Shahroud, for two sessions. The forms were completed through face to face interviews with mothers and some data were collected from health records for all infants who were born during 2010-2011. Interview sessions were held in the health centers. Before each interview, oral consents were obtained from the mothers and if they were willing to participate, the data collection forms were completed. The forms were filled out one year after the infants’ births. Protocol of the present study was approved by the Ethics Committee of Shahroud University of Medical Sciences, No. 9047, in 2010.

### 3.1. Data Analysis

Using principal component analysis for socioeconomic variables including education and job of the household's head, marital status, composition of the household members, and 13 items of home asset, a new variable was calculated. Based on median of this variable, two quintiles were made and the studied population was divided to two groups: high socioeconomic group (n = 2705) and low socioeconomic group (n = 592). The infant mortality rate was compared between the two socioeconomic groups to assess the inequality. Univariate regression was performed on variables affecting infant mortality. Multivariate logistic regression model was then constructed by entering all the statically significant variables (full model) and the less effective ones were excluded in a backward method. The final multiple regression model included infant's birth weight, mother's education, infant's nutrition type, and consanguinity of parents. These variables were entered in the Oaxaca decomposition model to investigate determinants of inequality in infants’ mortality. This method decomposed the gap between the outcome variables of the studied socioeconomic groups into two components: One component was related to the obvious differences of the investigated variables between the two groups, called explained or endowment component. The other component was related to the differences in the influence of the studied variables between the two groups, called unexplained or coefficient component (7). Stata version 11 and SPSS version 17 were used to implement the above statistical operations. Significance level of 0.05 (95% CI) was used in all the tests.

## 4. Results

In this study, 72 of 3297 infants were dead (21.8 per 1000 live births); 52 died during the neonatal period (72.2%) and 20 died between 29 days and one year of age (27.7%). The mean of the mothers' education in the alive and dead groups was 10.17 (95% CI: 6.32-14.02) and 8.93 (95% CI: 4.48-13.38) years, respectively. Low birth weight was reported in 8.9% of the alive group and 69.4% of the dead group (Table 1).

**Table 1.** Demographic Characteristic of the Study Participants ^a^

Independent Variables	Child Viability Status		P Value^b^
	Alive (n = 3225)	Dead (n = 72)	
**Child gender**			0.03
Girl	1565 (48.5)	26 (36.1)	-
Boy	1660 (51.4)	46 (63.8)	-
**Birth weight, g**			< 0.001
≥ 2500	2938 (91.1)	22 (30.5)	-
< 2500	287 (8.9)	50 (69.4)	-
**Infant nutrition type**			< 0.001
Exclusive breastfeeding	2067 (64.0)	27 (49.9)	-
Other nutrition types	1158 (35.9)	39 (59.0)	-
**Mother's age, y**	27.30 (21.78-32.82)	27.15 (21.73-32.57)	0.83
**Father's age, y**	31.44 (25.32-37.56)	30.25 (23.76-36.74)	0.10
**Mother's education, y**	10.17 (6.32-14.02)	8.93 (4.48-13.38)	< 0.001
**Father's education, y**	10.02 (6.13-13.91)	9.29 (4.63-13.95)	0.11
**Mother's job**			0.27
Housewife	2923 (90.6)	68 (94.4)	-
Employed	302 (9.3)	4 (5.5)	-
**Consanguinity of parents**			< 0.001
Positive	2382 (73.8)	37 (51.3)	-
Negative	843 (26.1)	35 (48.6)	-
**Living area**			0.03
Urban	2005 (62.1)	36 (50)	-
Rural	1220 (37.8)	36 (50)	-
**Socioeconomic status**			< 0.001
High	2659 (82.4)	46 (63.8)	-
Low	566 (17.5)	26 (36.1)	-
**Pregnancy intervals, y**	2.94 (0.84-6.72)	3.01 (0.74-6.76)	0.87
**Gravid**	1.80 (0.70-2.90)	1.93 (0.76-3.1)	0.82
**Prenatal care**			0.74
Positive	767 (24.7)	19 (26.3)	-
Negative	2428 (75.2)	53 (73.6)	-
**High risk pregnancy**			0.005
Positive	1870 (57.9)	25 (34.7)	-
Negative	1355 (42.0)	47 (65.2)	-
**Delivery type**			0.85
Normal vaginal delivery	1444(44.7)	33 (45.8)	-
Caesarean section	1781(55.2)	39 (54.1)	-

^a^ Data are presented as No. (%) or mean (95% CI).

^b^ Chi-square test was used for categorical variables and independent t-test was applied for continuous ones.

Results of univariate logistic regression model showed that infant’s gender and nutrition type, consanguinity of parents, birth weight, mother's education, high-risk pregnancy, socioeconomic status, and living area had significant effects on the infant mortality. Results of multivariate logistic regression model showed that the odds of death in infants who were breast-fed during the first six months was 2.27 times (95% CI: 1.23-3.88) lower than others and this odds for infants whose parents were relatives was 2.37 times (95% CI: 1.38-4.08) more and also for infants with low birth weights it was 21.60 times (95% CI: 12.54-37.21) more. Each year of increase in the mother’s education led to 0.92 decrease in the odds of mortality (95% CI: 0.86-0.99). Infant mortality rate was 15.1 in the high and 42.3 in the low socioeconomic group. Based on multivariate logistic regression model, odds of death in the low socioeconomic group was 1.91 times (95% CI: 1.07-3.42) more than the high socioeconomic one (Table 2).

**Table 2.** Effects of Independent Variables on Infant Mortality ^a^

Independent Variables	Crude Odds Ratio^b^	95% CI	P Value	Adjusted Odds Ratio^c^	95% CI	P Value
**Child gender**						
Female	1	-	-	1	-	-
Male	1.66	1.02-2.71	0.03	1.33	0.78-2.28	0.20
**Birth weight, g**						
≥ 2500	1	-	-	1	-	-
< 2500	23.26	13.88-38.97	< 0.001	21.60	12.54-37.21	< 0.001
**Infant nutrition type**						
Exclusive breastfeeding	1	-	-	1	-	-
Other nutrition types	2.57	1.57-4.23	< 0.001	2.27	1.23-3.88	0.03
**Mother's age, y**	0.99	0.95-1.03	0.82	-	-	-
**Father's age, y**	0.96	0.93-0.82	0.10	1	-	-
**Mother's education, y**	0.87	0.82-0.93	< 0.001	0.92	0.86-0.99	0.04
**Father's education, y**	0.95	0.89-1.01	0.11	-	-	-
**Mother's job**						
Housewife	1	-	-	-	-	-
Employed	0.56	0.20-1.57	0.27	-	-	-
**Consanguinity of parents**						
Positive	1	-	-	1	-	-
Negative	2.67	1.67-4.27	< 0.001	2.37	1.38-4.08	0.002
**Living area**						
Urban	1	-	-	1	-	-
Rural	1.64	1.02-2.62	0.03	1.12	0.63-1.97	0.60
**Socioeconomic status**						
High	1	-	-	1	-	-
Low	2.65	1.62-4.33	< 0.001	1.91	1.07-3.42	0.02
**Pregnancy intervals, y**	1.00	0.94-1.06	0.87	-	-	-
**Gravid**	1.01	0.83-1.24	0.86	-	-	-
**Prenatal care**						
Positive	1	-	-	-	-	-
Negative	0.91	0.53-1.55	0.74	-	-	-
**High-risk pregnancy**						
Positive	1	-	-	1	-	-
Negative	2.00	1.22-3.26	0.006	1.48	0.85-2.60	0.16
**Delivery type**						
NVD	1	-	-	-	-	-
C/S	0.95	0.59-1.53	0.85	-	-	-

^a^ Abbreviations: CI, confidence interval; C/S, caesarean section; NVD, normal vaginal delivery.

^b^ Univariate Logistic Regression.

^c^ Multivariate Logistic Regression.

The effective variables in multivariate logistic regression model including birth weight, infant's nutrition type, mother's education and consanguinity of parents, were entered in the Oaxaca decomposition model. The results of decomposition (Table 3) showed that the difference between infant mortality rates in the two groups was -27.1 in favor of the high socioeconomic group. Of this gap, -11.9 per 1000 (44% of the gap) was related to the differences in the variables measured in the two groups (explained component) and -15.2 per 1000 (56% of the gap) was related to the differences in the β coefficients and intercept between the two groups (unexplained component).

**Table 3.** Decomposition of the Gap in Infant Mortality Between the Two Socioeconomic Groups ^a^

Infant Mortality Rate and Reason	Prediction, Per 1000 live births	95% CI	P Value
**Prevalence in high socioeconomic group**	15.18	10.72 to 19.64	< 0.001
**Prevalence in low socioeconomic group**	42.30	27.48 to 57.11	< 0.001
**Differences**	-27.11	-11.64 to -42.58	0.001
**Due to endowments (explained)**			
Mother's education	-7.43	-3.23 to -11.62	0.001
Consanguinity of parents	-1.47	-0.10 to -2.84	0.03
Infant's nutrition type	-1.05	0.02 to -2.14	0.05
Birth weight	-1.94	-4.01 to 0.13	0.06
Total	-11.90	-16.86 to -6.94	< 0.001
**Due to coefficients (unexplained)**			
Mother's education	16.08	-52.41 to 84.58	0.64
Consanguinity of parents	-1.82	-17.51 to 13.85	0.81
Infant's nutrition type	-12.55	-55.54 to 30.44	0.56
Birth weight	-0.67	-8.05 to 6.71	0.85
Constant	-16.24	-80.98 to 48.49	0.62
Total	-15.21	-29.03 to -1.39	0.03

^a^ Abbreviation: CI, confidence interval.

## 5. Discussion

In this population-based study, infant mortality rate of the low socioeconomic group was almost two times higher than that of the high socioeconomic group. This highlights the obvious inequality in children's health, which deserves special attention. The results of decomposition showed that if conditions of the two groups were the same with regard to the studied variables, 44% of their inequality could be eliminated. The remaining 56% gap between the two groups was related to the unexplained component, which was caused by differences in regression coefficients, intercepts and variables, which were not investigated in this study. The coefficient of no variables in the unexplained component was significantly different between the two groups and other factors that were not checked in this study could influence this part. All the differences in variables were in favor of the high socioeconomic group. Among the mentioned variables, mother's education was the most contributing factor in the explained component (62% of the explained component and 27% of the total gap); thus, the low socioeconomic group was more sensitive to changes in mother's education. Other studies also showed that infant mortality rate was higher in low-educated mothers (18, 19). Our results indicated that infant mortality rate decreased as birth weight increased. This was in line with results of many other studies conducted across the world (20, 21). Low birth weight is caused by different factors such as mother's disease before pregnancy, pregnancy complications, mother's lifestyle, (20) mother’s age, prenatal care, mother's weight gain during pregnancy (22), and other variables, which can be determined and controlled to reduce infant mortality. Paying attention to these factors especially in the low socioeconomic groups, who may have less access to healthcare services, must be set as a top health priority.

Among other findings of the present study, also mentioned in other studies, is consanguinity of parents and its role in infants’ mortality (23, 24). It is probably due to increase in congenital abnormalities among these children. Therefore, any program that would promote awareness about the risks of congenital marriage can be helpful and special attention to genetic consultation would be effective and efficient. Our results indicated that mortality rate of infants who were exclusively breast-fed in the first six months of their life, was significantly lower than those who used different formulas or foods. This indicates the protective role of mother's milk against diseases and mortality, on which other studies have emphasized (25, 26). Therefore, infants' morbidity and mortality could be prevented by breastfeeding promotion, especially in low socioeconomic groups. The Oaxaca decomposition method has been used in different health fields around the world (19, 27-29). However, for child mortality, this method has only been used by Van de Poel et al. (30). In Iran, the only study that investigated the inequality in child mortality and decomposed the gap between different groups was conducted by Hosseinpour et al., but they employed the concentration index for decomposition (16).

### 5.1. Strengths and Limitations

One of the strengths of the present study was the high response rate (97%). Applying a new method for identifying determinants of inequality was another point of strength. One of the main limitations of this study was using health records to complete some parts of the data collection form. Due to the study design, it was not possible to make causal inference between the infant mortality rate and known determinants. Another limitation could be unavailability of some children.

In conclusion, there is considerable socioeconomic inequality regarding infant mortality. In the present study, mother's education was the most important factor causing inequality. Other factors such as infant's nutrition type, consanguinity of parents, and birth weight can play a role in this inequality. To minimize the inequality, policymakers at the healthcare sector must pay attention to the unexplained component in addition to assessing those which have been identified, and provide appropriate policies to deal with them. They also need to provide facilities for conducting more researches to realize the unexplained components.
